# Intranasal Vaccination with Murabutide Enhances Humoral and Mucosal Immune Responses to a Virus-Like Particle Vaccine

**DOI:** 10.1371/journal.pone.0041529

**Published:** 2012-07-25

**Authors:** Erin M. Jackson, Melissa M. Herbst-Kralovetz

**Affiliations:** Department of Basic Medical Sciences, College of Medicine-Phoenix, The University of Arizona, Phoenix, Arizona, United States of America; Instituto Butantan, Brazil

## Abstract

Murabutide (MB) is a synthetic immunomodulator recognized by the nucleotide-binding oligomerization domain-containing protein 2 (NOD2) receptor on mammalian cells. MB has previously been approved for testing in multiple human clinical trials to determine its value as an antiviral therapeutic, and as an adjuvant for injected vaccines. We have found a new use for this immunomodulator; it functions as a mucosal adjuvant that enhances immunogenicity of virus-like particles (VLP) administered intranasally. MB enhanced Norwalk virus (NV) VLP-specific IgG systemically and IgA production at distal mucosal sites following intranasal (IN) vaccination. A dose escalation study identified 100 µg as the optimal MB dosage in mice, based on the magnitude of VLP-specific IgG, IgG1, IgG2a and IgA production in serum and VLP-specific IgA production at distal mucosal sites. IN vaccination using VLP with MB was compared to IN delivery VLP with cholera toxin (CT) or gardiquimod (GARD) and to parenteral VLP delivery with alum; the MB groups were equivalent to CT and GARD and superior to alum in inducing mucosal immune responses and stimulated equivalent systemic VLP-specific antibodies. These data support the further testing of MB as a potent mucosal adjuvant for inducing robust and durable antibody responses to non-replicating subunit vaccines.

## Introduction

The majority of US FDA approved vaccines are administered parenterally (subcutaneous or intramuscular routes) and induce systemic immune responses (measured by serum antibody production). This systemic IgG may participate in local immune responses at distal mucosal sites, however with reduced efficacy relative to secretory IgA (sIgA). Since many pathogens gain entry through mucosal sites, efforts have been made to induce robust sIgA throughout the common mucosal immune system (CMIS) by mucosal vaccination. The development of these mucosal vaccines has been limited by the lack of mucosal adjuvants that are both safe and potent inducers of mucosal and systemic immune responses.

Most vaccines in use today are formulated with aluminum salts to enhance immunogenicity. Despite the long history of utilizing these aluminum salt formulations as adjuvants, the mechanism of action was not elucidated until several recent studies have implicated sensing by the nucleotide-binding domain leucine-rich repeat and pyrin domain containing receptor 3 (NLRP3) to mediate systemic immune responses characterized by the production of IgG1 and IgE [1], [2], [3], [4]. Furthermore, adjuvants containing alum, in the form of crystalline aluminum oxyhydroxide, amorphous aluminum hydroxyl-phosphate, or a formulation of anhydrous aluminum hydroxycarbonate (Imject® alum) differ from each other in the exact mechanisms that result in systemic immune responses to the target antigen [3].

The use of cholera toxin (CT) as a mucosal adjuvant is known to induce potent systemic and mucosal antigen-specific immune responses. However, its use in human clinical trials has resulted in high toxicity and is therefore unsuitable for use in humans [5], [6], [7]. Most work on the development of adjuvants has been focused on utilizing innate immunomodulators that trigger pattern recognition receptors (PRR), including toll-like receptors (TLRs) [5], . Our group has previously demonstrated the efficacy of TLR agonists as mucosal adjuvants, such as the TLR7 agonist, gardiquimod (GARD), in eliciting a robust mucosal immune response to a subunit antigen [8], [9]. In this work, we focus on triggering another PRR family, the nucleotide-binding oligomerization domain-containing protein 2 (NOD2) receptor using an immunomodulator called murabutide (MB). Few groups have studied NOD2 agonists as adjuvants [10], [11] and to our knowledge we are the first group to evaluate MB as a mucosal adjuvant for a virus-like particle (VLP)-based vaccine.

In 1974, MDP had been identified as an immunostimulant that induced non-specific immune responses to antigens [12], [13], [14]. MB, a synthetic derivative of the bacterial cell wall peptidoglycan muramyl dipeptide (MDP), was developed as a safe alternative to MDP for use as an immunomodulator, after MDP was found to be too toxic to be used as an adjuvant in humans [5], [13], [15]. MB possesses all of the immunomodulatory properties of its parent molecule, MDP, without the associated toxicity that includes pyrogenicity, somnogenicity, and acute and chronic inflammation, and has proven to be well tolerated in preclinical animal and human clinical trials [12], [16], [17], [18], [19], [20], [21], [22], [23].

MB, like MDP, contains the minimal necessary conserved structural motif of peptidoglycan to be recognized by the NOD2 receptor on host cells [12], [23], [24], [25], [26], [27]. NOD2 is a PRR that recognizes distinct pathogen associated molecular patterns (PAMPs) and results in stimulating mediators of inflammation [28], [29], [30]. Located on key antigen presenting cells (APCs) and T lymphocytes, NOD2 receptors play a critical role in host response to pathogens, specifically at mucosal surfaces where these receptors are less abundant [20], [29], [30], [31], [32], [33]. MB primarily targets these innate cells to stimulate non-specific resistance to pathogens, induce innate and adaptive immune responses through activation of APCs and cytokine production, and to enhance immunogenicity to target antigens [12], [23], [26], [27], [31], [32]. Previous hepatitis B and antiviral HIV-1 clinical trials and vaccine studies have shown that MB regulates host cell receptor expression, inhibits viral replication, and induces sustainable antigen-specific antibodies in response to antigens [12], [16], [17], [20], [21], [22], [23], [34], [35], [36].

Norwalk virus virus-like particles (NV VLP) can be used as a model mucosal subunit vaccine to test the adjuvanticity of novel immunopotentiators, formulations, or routes of vaccination [8], [9], [37], [38], [39]. Studies conducted as a component of product development for norovirus vaccines, have shown that mucosal vaccination with these NV VLP and adjuvants produce varying levels of VLP-specific antibodies that are functional as a result of blocking binding to histo-blood group antigens (HBGA), preclinically and in humans [40], [41], [42]. We hypothesize that activation of the NOD2 pathway by mucosal co-delivery of MB with VLP, would result in a robust mucosal and systemic immune response, equivalent to the response with the use of the mucosal adjuvant CT or GARD, whereas parenteral vaccination with alum as an adjuvant would only stimulate a systemic immune response. For our studies, the amplitude of systemic immune responses is measured as serum antibody titers, whereas IgA levels indicate mucosal immune response. Using NV VLP as our mucosal antigen and MB as our mucosal adjuvant, we performed a dose-ranging escalation study to identify the least effective MB dose required to induce both optimal systemic and mucosal antibody production.

## Materials and Methods

### NV VLP production

Recombinant NV VLP were expressed in *Nicotinia benthamiana* by Kentucky Bioprocessing (Owensboro, KY) and clarified leaf extracts were filtered and fractionated as previously described [8].

### Preparation of Murabutide, Cholera Toxin and Gardiquimod

MB stock solution of 10 mg/ml was prepared by resuspending 5 mg lyophilized murabutide powder (InvivoGen, San Diego, CA) in 500 µl sterile endotoxin free water and vortexed until fully resuspended. Stocks were stored long-term at −80°C and short-term at −20°C until use. Cholera toxin (List Biological Laboratories, Inc., Campbell, CA) and gardiquimod (InvivoGen) were prepared as previously described [8], [9].

### Mouse Vaccination

All animals were housed in accordance with American Association for Laboratory Animal Care (AALAC) standards, provided unlimited access to food and water, and all procedures and handling for this study were approved by the Animal Welfare Act and Arizona State University (ASU) Institutional Animal Care and Use committee (IACUC) (Assurance # A3217-01). Prior to vaccination, 5-week-old inbred female BALB/c mice (Charles River Laboratories International, Inc., Wilmington, MA) were randomly distributed into 8 groups (*n* = 7 per group) for study 1 and 3 groups for study 2 and allowed to acclimate for 1 week. In the first study, mice were vaccinated intranasally on day 0 and 21, as previously described [8] for liquid formulations containing 25 µg VLP alone, 25 µg VLP +25 µg MB, 25 µg VLP +100 µg MB, 25 µg VLP +250 µg MB, 250 µg MB alone, 25 µg VLP +1 µg CT (1 mg/ml in PBS, List Biological Laboratories, Inc.) which is a well characterized mucosal adjuvant, PBS alone as negative control, or 25 µg VLP + alum (ratio of 1∶1; Imject® Alum, Pierce Biotechnology, Rockford, IL) delivered subcutaneously. In the second study, mice were vaccinated intranasally on day 0 and day 21, as previously described [8] for liquid formulations containing 25 µg VLP alone, 25 µg VLP +200 µg MB, 25 µg VLP +25 µg GARD, or PBS alone as negative control. Mice were not anesthetized for vaccinations.

### Sample Collection

Serum samples, fecal pellets, and vaginal lavage samples were collected on day 0 prior to vaccination (preimmune), and on days 12, 21, 42, and 56 for study 1 and on day 0 prior to vaccination (preimmune), and on days 12, 21, 42, 56, 84, and 112, as previously described. All mice were humanely euthanized on day 56 (study 1) and day 112 (study 2) in accordance with the Animal Welfare Act and ASU IACUC. Uterine lavages were collected post-mortem by opening the abdominal cavity, fully excising each uterine horn, and flushing each horn with 200 µl PBS, as previously described [9]. Mucosal samples including salivary, nasal, and vaginal lavages were collected on day 56 (study 1) and day 112 (study 2) following euthanasia as previously described [8]. Splenocyte isolation was performed as previously described [8].

### NV-specific Enzyme-Linked ImmunoSorbent Assays

Insect cell-derived NV VLP were manufactured and purified as previously described [8]. ELISAs were performed using 10% (fecal and intestinal samples) or 5% (mucosal samples) dry milk in PBS for blocking and antibody dilutions of IgG (1∶5,000) (Southern Biotech, Birmingham, AL), IgG1 (1∶2,500) (Santa Cruz Biotechnology Inc., Santa Cruz, CA), IgG2a (1∶2,500) (Santa Cruz Biotechnology Inc.), or IgA (1∶5,000) (Sigma-Aldrich) as previously described [8]. Endpoint titers are reported as the reciprocal of the highest dilution that had an absorbance greater than or equal to 0.075 above the background.

### Splenocyte NV-specific Enzyme-Linked Immunospot Assays

Spleens were harvested following euthanization on day 112 as previously described [8]. ELISPOTs were performed as previously described [8]. Spots were counted with the ImmunoSpot S3B ELISPOT analyzer (Cellular Technology Ltd., Shaker Heights, OH) and expressed as the average number of spots per 1×10^7^ IgG secreting splenocytes.

### Statistical Analysis

Prism software (GraphPad, Inc., San Diego, CA) was used to graph and evaluate statistical comparisons of all data. NV VLP-specific IgA and IgG antibody titers are expressed as geometric mean titers (GMT) for each vaccination group at each time point. Samples from the PBS groups of mock-vaccinated mice were negative by ELISA (not shown). All responders and nonresponders were included in the computation of the GMT and negative samples were assigned the value of 1.0 for calculation purposes. The Kruskal-Wallis one-way analysis of variance (ANOVA) followed by a Dunn’s post-test was used to compare ELISA values at each time point between individual treatment groups versus PBS alone. One-way analysis of variance (ANOVA) followed by Bonferroni’s post-test was used to compare ELISPOT values between individual treatment groups versus PBS alone. Statistical significance was considered to be a *P* value of <0.05.

## Results

### VLP Codelivered with MB or Alum Elicit a Strong Systemic Immune Response

**Figure 1:**
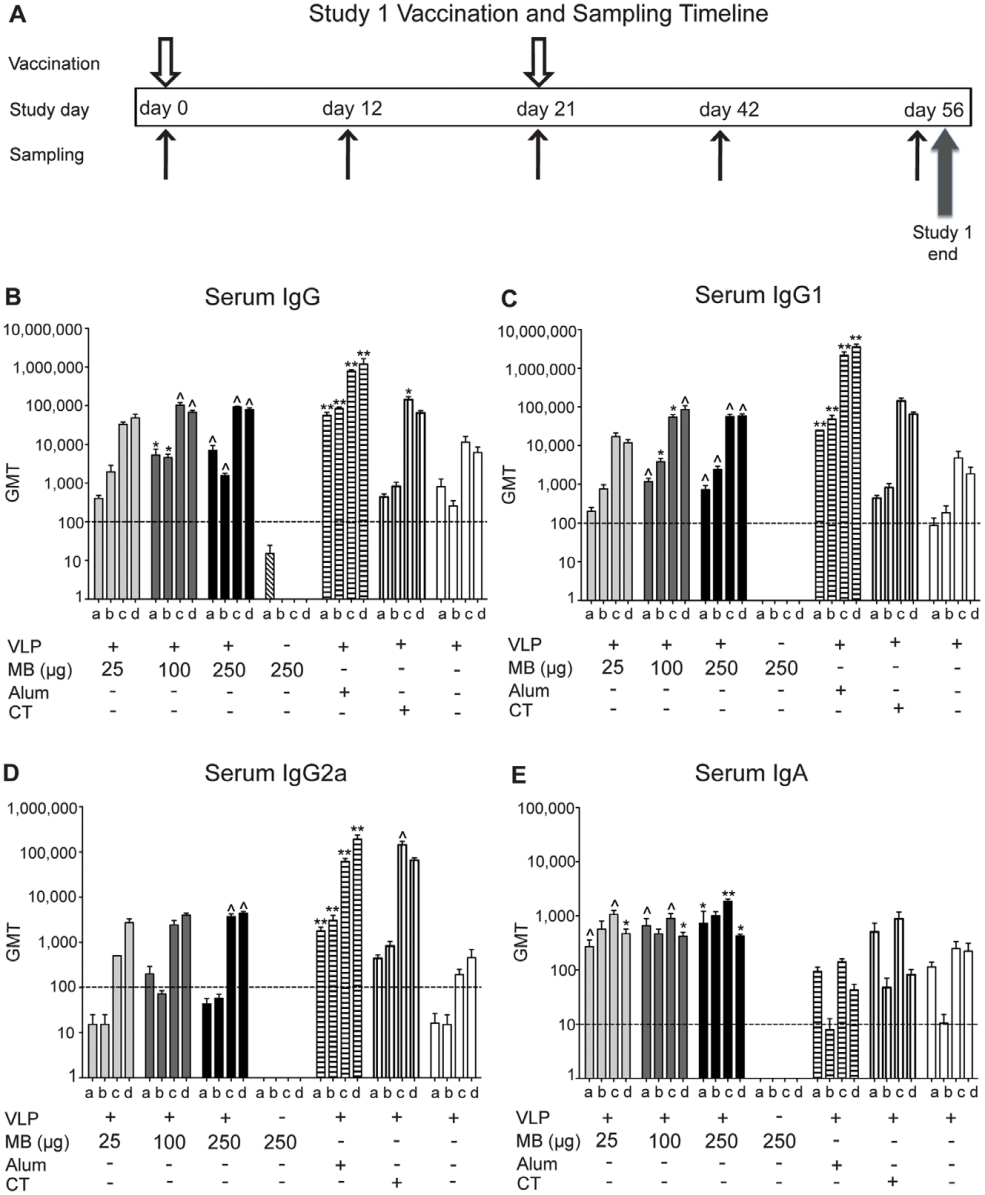
VLP vaccination and sampling schedule of mice and VLP-specific humoral immune response. (A) Timeline of study 1 vaccinations and sample collection days. Serum VLP-specific IgG (B), IgG1 (C), IgG2a (D), and IgA (E) production following IN delivery of VLP and MB (25 µg, 100 µg, or 250 µg), MB alone (250 µg), VLP and alum delivered subcutaneously, VLP and CT delivered IN, or VLP alone delivered IN on day 0 and day 21. Serum samples were collected on days 0 (not shown), 12 (a), 21 (b), 42 (c), and 56 (d) and analyzed for VLP-specific antibody production by ELISA and presented as the geometric mean titer (GMT). Error bars represent the standard error of the means. Values that were significantly different from the values for the PBS control group are shown as: ^Λ^P<0.05, *P<0.01, **P<0.001. Samples from the PBS group of mock-vaccinated mice were negative by ELISA (not shown). The horizontal hatched line indicates the limit of detection for the assay.

To evaluate the immunogenicity of MB as a mucosal adjuvant in a dose-escalation study (study 1), liquid formulations of VLP (25 µg) were administered intranasally on study day 0 and day 21 and compared to three doses of VLP and MB (25 µg, 100 µg, and 250 µg), VLP and CT, or delivered parenterally with alum (Fig. 1A). All vaccinations were well tolerated and no adverse reactions in the mice were observed. Serum samples collected from each vaccination group (n = 7 mice/group) were assayed for VLP-specific IgG, IgG1, IgG2a, and IgA antibodies by ELISA. Serum IgG, IgG1, IgG2a, and IgA titers increased following boosting on day 21 for all VLP-containing vaccination groups and remained elevated throughout the study (day 56) (Fig. 1 B-E). VLP groups codelivered with MB (100 µg and 250 µg), and VLP and alum produced significant levels of serum IgG by day 12 that continued for the duration of the study (P 0.05–0.001), while VLP and CT elicited significant levels by day 42, compared to PBS controls (Fig. 1B). The alum group produced significantly higher levels of serum IgG compared to PBS controls and VLP alone for the duration of the study (P<0.05). Alum and VLP, and produced significantly higher titers at day 42 than VLP and MB (25 µg), but the levels were not significant at other time points compared to PBS or compared to the remaining VLP groups at any time point in the study (Fig. 1B). Serum IgG1 titers were significant at all time-points for VLP and MB (100 µg and 250 µg) and VLP and alum groups relative to PBS control (P<0.05–0.001). However, levels produced from all VLP and MB (25 µg, 100 µg, and 250 µg) groups compared to VLP and alum group were not significant at all time-points in the study (Fig. 1C). Serum IgG1 titers following boosting increased by 23-, 14-, and 23-fold for the VLP codelivered with MB (25 µg, 100 µg, and 250 µg) groups, respectively, while titers following boosting increased by 44- fold for the VLP and alum group, and 25-fold for the VLP alone group, and all levels remained consistently elevated throughout the duration of the study (day 56) (Fig. 1C). Serum IgG1 production from VLP and MB (25 µg), VLP and CT, and VLP alone groups were all similarly elevated in comparison to PBS group, but did not reach statistical significance. Serum IgG2a titers for all VLP-containing groups increased following boosting and remained elevated throughout the study, while levels from the VLP and alum group produced significant levels at all time-points, and the VLP codelivered with MB (250 µg) or VLP with CT groups produced significant levels following boosting, compared to PBS control (Fig. 1D). All VLP and MB (25 µg, 100 µg, and 250 µg) groups produced higher levels of serum IgA compared to VLP and alum, VLP and CT, or VLP alone (Fig. 1E). Serum IgA titers for all MB groups increased following boosting on day 21 and remained significantly elevated for the duration of the study, compared to mock-vaccinated mice (P<0.001). There was no significant difference in serum IgA titers produced by VLP and alum, VLP and CT, or VLP alone compared to PBS control-vaccinated mice (Fig. 1E).

The antigen to adjuvant dose-response was evaluated with intranasal delivery of VLP (25 µg) codelivered with 25 µg, 100 µg, or 250 µg of MB. Following intranasal vaccination with VLP and MB (100 µg or 250 µg), mice (n = 7 each group) produced VLP-specific serum IgG and IgG1 by day 12 that were significantly higher than mock-vaccinated mice, and remained elevated for the duration of the study (Fig. 1B–C). The lowest dose of MB (25 µg) co-delivered with VLP did not significantly induce levels of VLP-specific IgG, IgG1 or IgG2a antibodies relative to mock control (Figure 1). Serum IgG2a titers were significantly higher than PBS control-vaccinated mice for VLP and alum by day 12 and remained elevated throughout the study, while VLP and MB (250 µg) and VLP and CT produced significant levels by day 42, compared to PBS control-vaccinated mice (Fig. 1D). All VLP and MB (25 µg, 100 µg, and 250 µg) groups produced significant levels of serum IgA compared to PBS control-vaccinated mice at all time-points in the study, and VLP and alum, VLP and CT, and VLP alone groups produced elevated serum IgA levels, but were not statistically significant compared to PBS control-vaccinated mice (Fig. 1E).

### Intranasal Vaccination with MB is Superior to Alum in Producing a VLP-specific Mucosal Immune Response in the Gastrointestinal Tract

**Figure 2:**
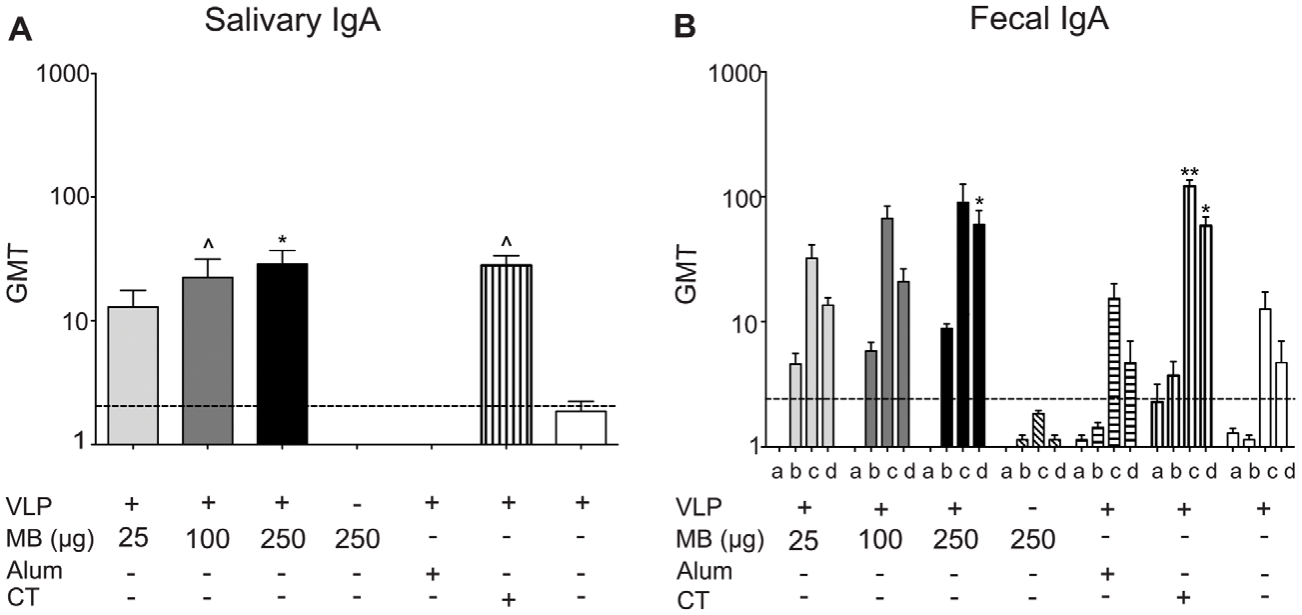
VLP-specific IgA production in the gastrointestinal tract. Mucosal IgA production was quantified in two sites within the gastrointestinal tract following IN delivery of VLP and MB (25 µg, 100 µg, or 250 µg), MB alone (250 µg), VLP and alum delivered subcutaneously, VLP and CT delivered IN, or VLP alone delivered IN on day 0 and day 21. (A) Salivary samples were collected from mice at day 56 and analyzed for VLP-specific IgA production by ELISA and presented as the geometric mean titer (GMT). (B) Fecal pellets were collected from mice on days 0 (not shown), 12 (a), 21 (b), 42 (c), and 56 (d) and analyzed for VLP-specific IgA by ELISA and presented as the geometric mean titer (GMT). Error bars represent the standard error of the means. Values that were significantly different from the values for the PBS control group are shown as: ^Λ^P<0.05, *P<0.01, **P<0.001. Samples from the PBS group of mock-vaccinated mice were negative by ELISA (not shown). The horizontal hatched line indicates the limit of detection for the assay.

We evaluated the ability of VLP codelivered with MB (25–250 µg), VLP with alum, VLP and CT, and VLP alone to induce VLP-specific IgA antibodies in the saliva and fecal extracts. Intranasal vaccination with VLP codelivered with MB at all dosages (25–250 µg) produced equivalent levels of VLP-specific salivary IgA relative to VLP and CT, and higher and more sustainable levels of VLP-specific salivary IgA compared to levels produced by VLP alone and by antigen delivered with alum (Fig. 2A). VLP delivered with the higher doses of MB (100 µg and 250 µg) and VLP and CT produced significantly higher levels of VLP-specific salivary IgA than mock-vaccinated groups (P<0.05, P<0.01, P<0.05 respectively).

Mice vaccinated with VLP codelivered with MB at all dosages tested and VLP and CT produced more sustainable levels of VLP-specific fecal IgA antibodies compared to mice vaccinated with either antigen alone or antigen and alum (Fig. 2B). Mice vaccinated with antigen and MB (250 µg) produced significant levels of VLP-specific fecal IgA antibodies by day 56 (P<0.01) and VLP and CT by day 42 (P<0.001) that remained elevated throughout the study (P<0.01), compared to PBS control-vaccinated mice.

### Intranasal Vaccination with MB is Superior to Parenteral Delivery with Alum in Inducing a VLP-specific Mucosal Immune Response at Distal Sites in the CMIS

#### (i) Respiratory tract

**Figure 3:**
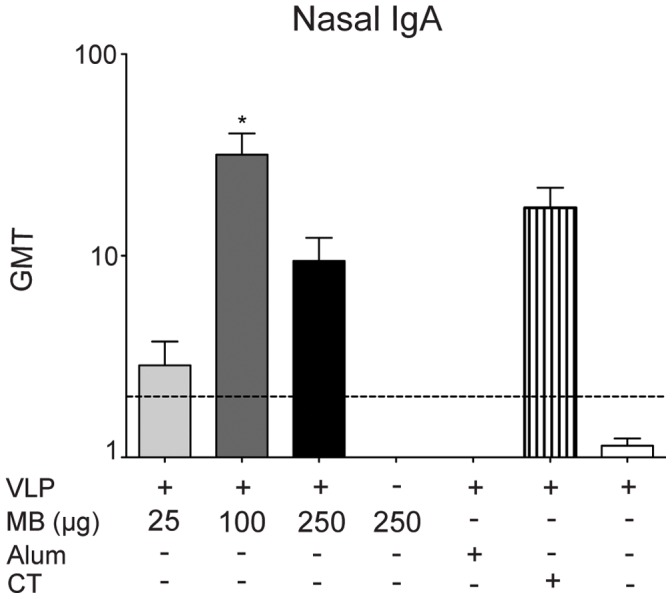
VLP-specific IgA production in the respiratory tract. Mucosal IgA production was quantified in the respiratory tract following IN delivery of VLP and MB (25 µg, 100 µg, or 250 µg), MB alone (250 µg), VLP and alum delivered subcutaneously, VLP and CT delivered IN, or VLP alone delivered IN on day 0 and day 21. Nasal secretions were collected from mice on day 56, and analyzed for VLP-specific IgA by ELISA and presented as the geometric mean titer (GMT). Error bars represent the standard error of the means. Values that were significantly different from the values for the PBS control group are shown as: ^Λ^P<0.05, *P<0.01, **P<0.001. Samples from the PBS group of mock-vaccinated mice were negative by ELISA (not shown). The horizontal hatched line indicates the limit of detection for the assay.

As the respiratory tract is the primary site of vaccination, we evaluated the ability of VLP codelivered with MB (25 µg, 100 µg, and 250 µg) compared to VLP and CT, VLP alone, and VLP delivered parenterally with alum to induce production of VLP-specific IgA antibodies in the nasal cavity (Fig. 3). VLP-specific production of nasal IgA was elevated in all groups vaccinated with MB (25 µg, 100 µg, and 250 µg) and VLP and CT compared to PBS control-vaccinated mice. The strongest response was elicited from the VLP and 100 µg MB group (P<0.01). There was no significant difference between the VLP alone and the VLP with alum groups in production of nasal IgA relative to PBS control-vaccinated groups.

#### (ii) Reproductive tract (vaginal and uterine)

**Figure 4:**
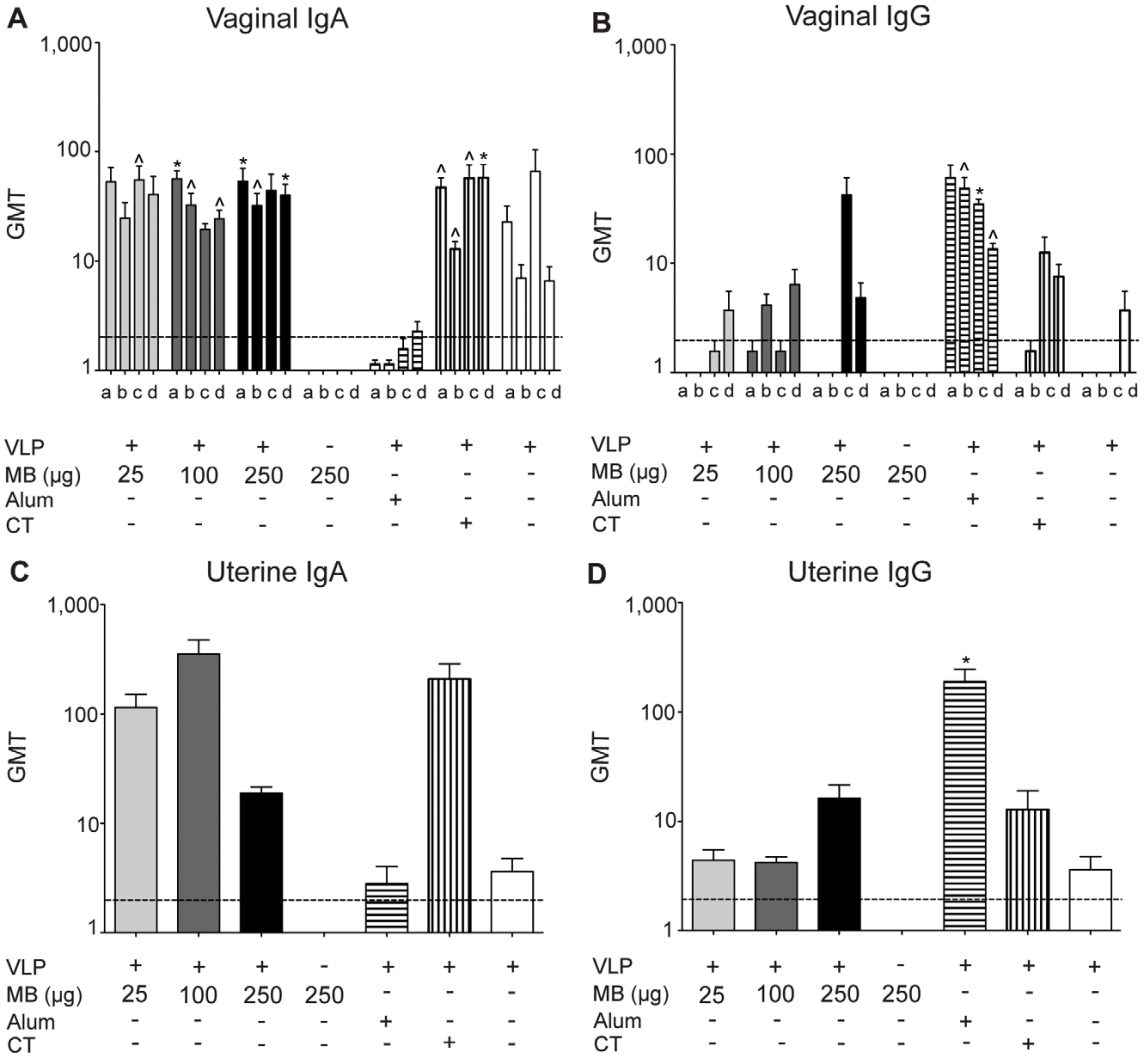
VLP-specific IgA and IgG production in the reproductive tract. Antibody levels were quantified in two sites in the reproductive tract following IN delivery of VLP and MB (25 µg, 100 µg, or 250 µg), MB alone (250 µg), VLP and alum delivered subcutaneously, VLP and CT delivered IN, or VLP alone delivered IN on day 0 and day 21. Vaginal lavages were collected from mice on days 0 (not shown), 12 (a), 21 (b), 42 (c), and 56 (d) and analyzed for VLP-specific IgA (A) and IgG (B) by ELISA. Uterine lavages were collected from mice on day 56 and analyzed for VLP-specific IgA (C) and IgG (D) by ELISA. Data is presented as the geometric mean titer (GMT). Error bars represent the standard errors of the means. Values that were significantly different from the values for the PBS control group are shown as: ^Λ^P<0.05, *P<0.01, **P<0.001. Samples from the PBS group of mock-vaccinated mice were negative by ELISA (not shown). The horizontal hatched line indicates the limit of detection for the assay.

As an additional measurement of mucosal immunity in the CMIS, we evaluated antibody production in the reproductive tract. Mice vaccinated with NV VLP codelivered with MB (25 µg, 100 µg, and 250 µg) and VLP and CT produced significant levels of NV VLP-specific vaginal IgA titers by day 12 and 21 and remained elevated throughout the study (P<0.05–0.01) (Fig. 4A). The alum group produced the lowest levels of VLP-specific vaginal IgA (Fig. 4A). VLP-specific vaginal IgG antibody levels were slightly elevated in the antigen delivered with MB (25 µg, 100 µg, and 250 µg) groups, VLP and CT, and VLP alone, compared to PBS control-vaccinated (Fig. 4B). The alum-containing group exhibited significant levels of VLP-specific vaginal IgG (P<0.01) by day 21, however the levels declined consistently for the remainder of the study (Fig. 4B).

Vaccination with VLP and MB (25 µg, 100 µg, and 250 µg) and VLP and CT resulted in high levels of uterine IgA compared to PBS control- vaccinated groups and were 3- and 4- fold higher than levels produced by the alum-containing group or VLP alone, respectively (Fig. 4C). Uterine VLP-specific IgG levels were significantly elevated in the VLP and alum group (P<0.01) and were elevated in all other VLP-containing vaccination compared to PBS control-vaccinated mice (Fig. 4E).

### Intranasal Vaccination with NV VLP Codelivered with MB Produce an Extended VLP- specific Systemic and Mucosal Immune Response

**Figure 5:**
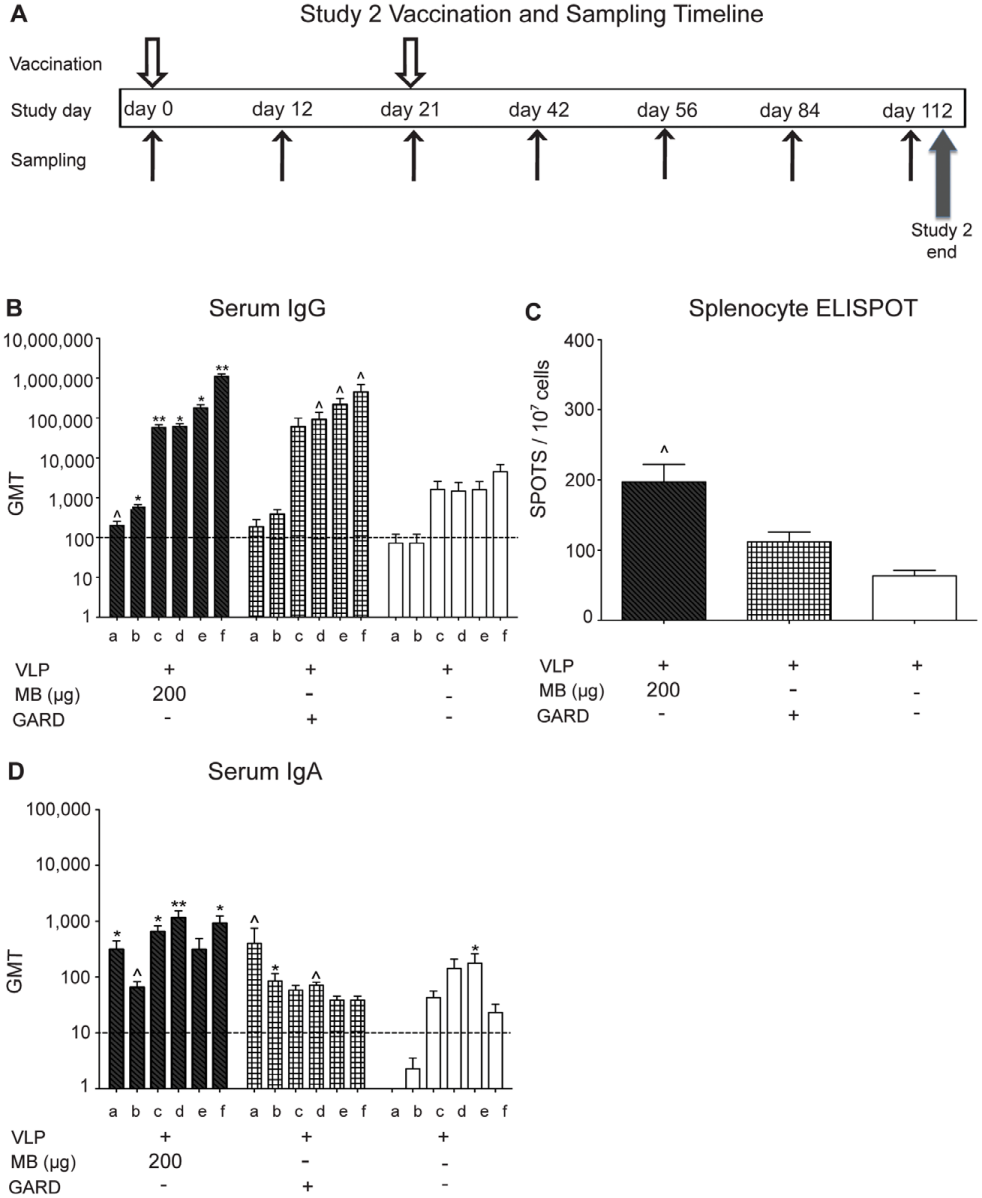
MB induces a sustained systemic immune response. (A) Timeline of study 2 vaccinations and sample collection days. The humoral immune response was measured and following IN delivery of VLP and MB (200 µg), VLP and GARD (25 µg), or VLP alone on day 0 and day 21 in an extended study (day 112). (B) Serum samples were collected on days 0 (not shown), 12 (a), 21 (b), 42 (c), 56 (d), 84 (e), and 112 (f), analyzed for VLP-specific IgG by ELISA and presented as the geometric mean titer (GMT). (C) On day 112, splenocytes were isolated and pooled into 3 groups to determine the frequency of VLP-specific IgG-secreting cells by ELISPOT. (D) Serum samples were collected on days 0 (not shown), 12 (a), 21 (b), 42 (c), 56 (d), 84 (e), and 112 (f), analyzed for VLP-specific IgA by ELISA and presented as the geometric mean titer (GMT). Values that were significantly different from the values for the PBS control group are shown as: ^Λ^P<0.05, *P<0.01, **P<0.001. Samples from the PBS group of mock-vaccinated mice were negative by ELISA (not shown). The horizontal hatched line indicates the limit of detection for the assay.

To determine the efficacy of MB as an adjuvant in eliciting an extended systemic and mucosal immune response, a parallel treatment study was conducted for 16 weeks (twice the length of the first study) and a time point used to evaluate long-term antibody responses [43], [44], [45], [46]. Study #2 contained four vaccination groups consisting of NV VLP (25 µg) delivered IN with a high dose of MB (200 µg) (n = 7), VLP (25 µg) with GARD (25 µg), VLP alone (25 µg) (n = 7 ), or PBS control- vaccinated delivery of PBS (n = 7). Serum IgG titers produced from the antigen with MB (200 µg) group were significantly higher than PBS control-vaccinated mice from day 12 (P<0.05) and continued for the duration of the study at day 112 (P<0.001) (Fig. 5B). VLP and GARD induced significant (P<0.05) levels of serum IgG production following boosting. The frequency of antigen-specific IgG secreting splenocytes in the VLP with GARD was elevated and the MB (200 µg)-containing group was significantly (P<0.05) elevated relative to the VLP alone group and the PBS group (Fig. 5C). Serum IgA titers from the VLP and MB (200 µg) group were significantly higher than PBS control-vaccinated mice (P<0.01–P<0.001) at day 12 and continued for the duration of the study (d112). Serum IgA production in VLP and GARD was significantly high beginning at day 12 (P<0.05) and decreased over the course of the study (Fig. 5D). The VLP and MB (200 µg) group showed a significant increase in serum IgA titers from day 12 to day 42 following boosting on day 21 (P<0.01–P<0.001) (Fig. 5D).

**Figure 6:**
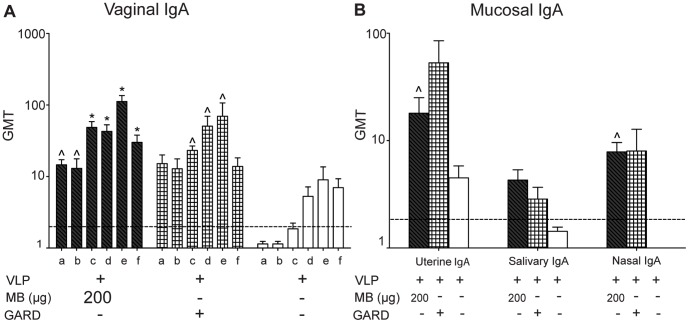
MB induces a sustained mucosal immune response at distal mucosal sites. VLP-specific antibody production was measured at reproductive, oral, and respiratory sites following IN delivery of VLP and MB (200 µg), VLP and GARD (25 µg), or VLP alone on day 0 and day 21 in an extended study (day 112). (A) Vaginal lavages were collected on days 0 (not shown), 12 (a), 21 (b), 42 (c), 56 (d), 84 (e), and 112 (f) and analyzed for VLP-specific IgA by ELISA. (B) Uterine, salivary, and nasal samples were collected on day 112 and analyzed for VLP-specific IgA by ELISA. Values that were significantly different from the values for the PBS control group are shown as: ^Λ^P<0.05, *P<0.01, **P<0.001. Samples from the PBS group of mock-vaccinated mice were negative by ELISA (not shown). The horizontal hatched line indicates the limit of detection for the assay.

Production of VLP-specific vaginal IgA in the VLP with MB (200 µg) group was significantly elevated throughout the duration of the study (day 112) relative to the PBS control-vaccinated group (P<0.01) (Fig. 6A). VLP with GARD also induced significantly (P<0.05) elevated levels of vaginal IgA production from days 42–84. The VLP alone group failed to produce vaginal IgA levels that were significant compared to the PBS control-vaccinated group. The antigen codelivered with MB (200 µg) or GARD resulted in 4-fold and 12-fold higher levels of uterine IgA than VLP alone, respectively, and were significantly higher than the PBS control-vaccinated group (P<0.05) (Fig. 6B). Salivary IgA production was 4-fold higher in the VLP and MB (200 µg) group, and 3-fold higher in the GARD group compared to VLP alone and PBS (Fig. 6B). Nasal IgA levels were significant at 8-fold higher in both the VLP and MB (200 µg) and GARD groups relative to VLP alone or PBS control-vaccinated mice (P<0.05) (Fig. 6B).

## Discussion

Subunit vaccines almost always require adjuvants to achieve robust and sustainable immune responses. However, many potent mucosal adjuvants, such as CT, may induce severe and toxic side effects, rendering them unsuitable for use in humans, while other adjuvants elicit only systemic immune responses [47]. Therefore, it is essential that an ideal mucosal adjuvant be well tolerated by the recipient, as well as capable of inducing durable and robust mucosal and systemic immune responses to the target antigen.

Alum is one of the few adjuvants approved by the FDA for clinical use and is a potent adjuvant for induction of systemic IgG responses in a variety of vaccines [5], [48]. In our first study, parenteral administration of VLP with alum induced a strong serum IgG and IgG isotype response. Vaginal and uterine IgG levels produced by VLP with alum were also significantly higher than PBS control-vaccinated mice or those vaccinated with NV VLP alone. This is an expected outcome as this indicates transcytosis of serum antibody to the reproductive mucosa [49], [50], [51]. All IgA antibody responses induced by the parenteral vaccination with VLP plus alum were comparable to PBS control-vaccinated groups, with the exception of fecal IgA, which was comparable to the response generated by VLP administration alone. The lack of stimulation of IgA production following parenteral vaccination with alum was not an unexpected finding. It is well documented that this route of administration combined with alum generates predominantly systemic immune responses, and IgA levels correlate to mucosal responses [47]. Taken together, this data demonstrates that while alum is effective at eliciting a strong systemic immune response to NV VLP, it induces little IgA and therefore may be a poor choice as an adjuvant for a vaccine requiring a strong mucosal immune response.

Our data provide evidence that murabutide is a mucosal adjuvant since it significantly enhances the induction and sustained levels of serum IgA production in mice following vaccination with NV VLPs (Fig. 1 and 5). Furthermore, serum IgA production in all VLP + MB containing groups was higher than titers produced by parenteral delivery with VLP and alum or intranasal delivery with the gold-standard mucosal adjuvant CT (Fig. 1E). This observation about murabutide may be relevant to studies by others who are evaluating delivery strategies for norovirus vaccines; in a recent human norovirus challenge study, a NV-specific IgA seroresponse in vaccine recipients correlated with protection against illness and infection when using an intranasal route of vaccination [40].

Our first study tested the ability of MB, delivered with NV VLP by IN vaccination, to elicit a more robust and durable systemic and mucosal immune response than that produced by vaccination with VLP alone, by parenteral vaccination with VLP plus alum or by codelivery with the mucosal adjuvant CT (Fig. 1–4) This dose-ranging study was performed with three escalating doses of MB to determine the optimal dose for induction of antigen-specific systemic and mucosal antibody responses. VLP co-delivered IN with MB (25 µg, 100 µg, and 250 µg) all resulted in the high titers of serum IgG, IgG1, and IgG2 compared to PBS control-vaccinated mice or VLP alone (Fig.1). Serum IgG1, IgG2a, and IgA titers were induced in MB-containing groups, suggesting a mixed Th1/Th2 response to VLP, similar to titers induced by CT (Fig. 1) In addition to enhancing systemic immunity, IN codelivery of VLP with MB significantly and consistently enhanced mucosal IgA responses in the reproductive, gastrointestinal, and respiratory tracts of vaccinated mice similar to CT (Fig. 2 to 4). The lowest dose of MB (25 µg) was not effective when codelivered with VLP at inducing significantly elevated levels of systemic or mucosal antibody. Our data indicate that vaccination with 25 µg VLP co-delivered with 100 µg or 250 µg of MB induce a more robust mucosal response than is produced by VLP alone and that mucosal site antigen-specific antibodies (IgA) were maintained at high levels for the duration of the study. These doses of MB co-delivered with VLP produced the most consistent antibody responses of VLP and MB containing groups and our data suggest this is the lowest effective dose range tested.

In our second study, we demonstrate that IN vaccination of VLP codelivered with MB not only elicits significant VLP-specific systemic and mucosal responses compared to VLP alone or PBS control-immunized mice, but the responses were sustained throughout this longer-term study (to day 112) (Fig. 5–6). Furthermore, IN vaccination of VLP with MB elicits antigen-specific systemic and mucosal immune responses similar to those produced by the previously tested mucosal adjuvant TLR7 agonist GARD [8]. We did not evaluate the functional activity of the antibodies, however others have show that antibodies produced in response to vaccination or experimental infection, in humans, with similar norovirus VLP-based vaccines are functional and block HBGA binding [40], [41], [42].

IN vaccination with MB induces robust VLP-specific systemic and mucosal responses. The enhanced systemic and mucosal immune responses elicited by the co-delivery of MB with VLP were sustained for the duration of both studies and no adverse effects were observed in mice following intranasal MB administration. We have demonstrated superior induction of mucosal antibodies (IgA) after IN vaccination with NV VLP with MB in comparison to parenteral delivery of the VLP with alum or IN delivery of VLP alone and equivalent mucosal IgA induction to IN codelivery of VLP with CT or GARD. Furthermore, sustainability of the immune response generated by vaccination using MB as a mucosal adjuvant, and the prior history of using MB in human clinical trials, make this immunomodulator an attractive candidate adjuvant for other subunit antigens and/or vaccine formulations for mucosal immunization.
